# Sequencing of bovine herpesvirus 4 v.test strain reveals important genome features

**DOI:** 10.1186/1743-422X-8-406

**Published:** 2011-08-16

**Authors:** Leonor Palmeira, Bénédicte Machiels, Céline Lété, Alain Vanderplasschen, Laurent Gillet

**Affiliations:** 1 Immunology-Vaccinology (B43b), Department of Infectious and Parasitic Diseases (B43b), Faculty of Veterinary Medicine, University of Liège, B-4000 Liège, Belgium

## Abstract

**Background:**

Bovine herpesvirus 4 (BoHV-4) is a useful model for the human pathogenic gammaherpesviruses Epstein-Barr virus and Kaposi's Sarcoma-associated Herpesvirus. Although genome manipulations of this virus have been greatly facilitated by the cloning of the BoHV-4 V.test strain as a Bacterial Artificial Chromosome (BAC), the lack of a complete genome sequence for this strain limits its experimental use.

**Methods:**

In this study, we have determined the complete sequence of BoHV-4 V.test strain by a pyrosequencing approach.

**Results:**

The long unique coding region (LUR) consists of 108,241 bp encoding at least 79 open reading frames and is flanked by several polyrepetitive DNA units (prDNA). As previously suggested, we showed that the prDNA unit located at the left prDNA-LUR junction (prDNA-G) differs from the other prDNA units (prDNA-inner). Namely, the prDNA-G unit lacks the conserved *pac-2 *cleavage and packaging signal in its right terminal region. Based on the mechanisms of cleavage and packaging of herpesvirus genomes, this feature implies that only genomes bearing left and right end prDNA units are encapsulated into virions.

**Conclusions:**

In this study, we have determined the complete genome sequence of the BAC-cloned BoHV-4 V.test strain and identified genome organization features that could be important in other herpesviruses.

## Background

Gammaherpesviruses are archetypal persistent viruses which are ubiquitous in both human and animal populations. The human gammaherpesviruses, Epstein-Barr virus (EBV) and Kaposi's Sarcoma-associated Herpesvirus (KSHV), infect respectively some 90% [1] and 30% [2] of human populations and cause several cancers [2,3]. Although much effort has been invested on these viruses, studies of EBV or KSHV are difficult to perform directly because these viruses show limited lytic growth *in vitro *and have no well-established *in vivo *infection model. Related animal gammaherpesviruses are therefore an important source of information.

Bovine herpesvirus 4 (BoHV-4) belongs to the *Gammaherpesvirinae *subfamily, and to the *Rhadinovirus *genus [4]. Similarly to its human counterparts, BoHV-4 was found to be widespread in all bovine populations and to persist in the vast majority of individuals as a lifelong, asymptomatic infection [5]. However, in some circumstances, BoHV-4 has been associated with various clinical symptoms such as skin lesions, respiratory diseases, metritis, malignant catarrhal fever or tumors [5].

The virus was first isolated in Europe by Bartha *et al*. from calves with respiratory diseases [6] and later in North America by Mohanty *et al*. [7]. Besides cattle, BoHV-4 has also been detected in a variety of ruminants. In particular, BoHV-4 seems to be highly prevalent among wild African buffalo (*Syncerus caffer*) which could be considered as the natural reservoir of the virus [8-10]. Overall, more than 40 BoHV-4 strains have been isolated across the world. These strains can be classified in three groups: the European strains (or Movar 33/63-like strains), the American strains (or DN 599-like strains) and the African buffalo strains [9].

It is estimated that the taurine and buffalo strains diverged around 730,000 years ago [9] and that the European and North American clades diverged around 260,000 years ago [9]. The genome of the BoHV-4 66-p-347 North American strain has entirely been sequenced [11]. However, the BAC-cloned reference strain V.test [12,13] belongs to the European clade [9,14]. Previous studies suggested that the BoHV-4 V-test strain contains regions of high dissimilarity compared to the BoHV-4 66-p-347 strain. Indeed, the nucleotide identity between the two strains has been previously measured to be as low as 88% on the BORFB2 region [11]. However, the lack of a complete genomic sequence for the V.test strain prevents from drawing a general view concerning this divergence level. Therefore, the low quality of the genomic information hampers the use of the BAC-cloned BoHV-4 V.test strain as a good model for studying gammaherpesvirus biology. In this study, we have determined the genomic sequence of the BoHV-4 V.test strain and analyzed its overall differences with the available sequence of the BoHV-4 66-p-347 strain [11,15]. The results obtained highlighted important differences between BoHV-4 66-p-347 and V.test strains. Moreover complete sequencing of the BoHV-4 V.test strain also revealed genome features potentially important in other herpesviruses.

## Methods

### BAC sequencing

BAC DNA was purified using Qiagen large-construct kit as described by the manufacturer. The complete BAC cloned viral genome of BoHV-4 V.test strain was determined by pyrosequencing using the 454 GS FLX Titanium (Roche) high-throughput sequencer and resulted in 48,967 reads of an average read length of 265 nucleotides and a total of 12,997,275 bases. A targeted ABI-Sanger sequencing of fragments of the prDNA region was also conducted using the primers listed in Table 1. The raw 454 data has been deposited in the NCBI Sequence Read Archive (SRA) database with accession number SRA037246.

**Table 1:** Primers used in this study

name	Sequence	Coordinates according to Genbank
Bo1 Fwd	5'- ATGGAGGGTGATGGATTCATG-3'	460-440^a^
Bo1 Rev	5'- TTAAGGCCTCATTCCAGGAAG-3'	272-292^a^
Bo5 Fwd	5'- GCTACAGAAAATGGCCAGTAAAG-3'	20366-20342^a^
Bo5 Rev	5'- TCATGTCCTGAGTGGGTCTATG-3'	19170-19191^a^
Bo6 Fwd	5'- ATGGTCATCCTAAATGCTCAAG -3'	20297-20318^a^
Bo6 Rev	5'- TCACCTAGTGTTGCAACCCC -3'	20497-20478^a^
Bo7 Fwd	5'- ATGGAGACAATTTCCATAAACTG -3'	20994-20972^a^
Bo7 Rev	5'- CTAGCTGGGGTAGAGTGATC -3'	20671-20690^a^
ORF67.5 Fwd	5'- ATGGCTGATGGTGATGTTTTAG -3'	93144-93123^a^
ORF67.5 Rev	5'- TCAATGTTTGTCCAGAGCACT -3'	92881-92901^a^
Bo12 Fwd	5'- ATGGGGGCGCTATTTGGGC -3'	97442-97460^a^
Bo12 Rev	5'- TCAACTGATGAAACCCACCC -3'	97525-97506^a^
Bo13 Fwd	5'- ATGCGTCTCGATGGCAAGC -3'	98838-98856^a^
Bo13 Rev	5'- CTATGGTTGTTTTTTAAAGAAAATC -3'	98981-98957^a^
ORF75 Fwd	5'- ATGTATCCCAGATACAGTAACA -3'	103606-103585^a^
ORF75 Rev	5'- TTACATTTTATTTTTCAGACACCA -3'	100274-100297^a^
prDNA Fwd 1	5'- GGAGCCCAAAACCAAAAGAG -3'	870-889^b^
prDNA Rev 1	5'- CTCTTTTGGTTTTGGGCTCC -3'	889-870^b^
prDNA Fwd 2	5'- CGTAGGCCTCACATTCAGC -3'	908-926^b^
prDNA Rev 2	5'- GCTGAATGTGAGGCCTACG -3'	926-908^b^
prDNA Fwd 3	5'- CGAGAGATGGTTCTTGCACA -3'	940-959^b^
prDNA Rev 3	5'- TGTGCAAGAACCATCTCTCG -3'	959-940^b^
BAC Rev	5'- TTGCCAATCCCAAAAAGAAG -3'	9859-9878^c^

^a ^according to Genbank JN133502 sequence (BoHV-4 V.test strain long unique region)

^b ^according to Genbank JN133504 sequence (BoHV-4 V.test strain prDNA inner region)

^c ^according to Genbank AY665170.1 sequence (pBeloBAC modified)

### BoHV-4 genome LUR assembly

The reads were *de novo *assembled with gsAssembler (Roche), where the *E. coli *genome was used as a contaminant to filter out cellular reads [16]. The filtering removed 1,167 contaminant cellular reads. The *de novo *assembly yielded 11 contigs which were subsequently BLASTed against 66-p-347's long unique region (LUR) and polyrepetitive DNA (prDNA) -accession numbers NC_002665 and AF092919- to define their relative positions [17]. Contigs were assembled into a large scaffold using two previously published V.test sequences (accession numbers Z46380 and Z46385 [18]) overlapping contig borders. A careful comparison of the bordering contigs with the previously sequenced fragments showed a high percent identity (> 99.99%). After verification of the quality of the assembly, the BAC sequence was removed and the genome sequence was annotated as detailed hereunder.

### BoHV-4 genome prDNA assembly

The prDNA was determined by a hybrid 454/ABI-Sanger strategy where 17 ABI-Sanger fragments of prDNA were *de novo *assembled with the 454 reads. Briefly, in order to correctly assemble the prDNA and to disentangle different prDNA units, this second *de novo *assembly was optimized for highly repetitive segments using MIRA [19]. 454 reads and quality information were extracted from the raw .sff file with 'sff_extract'. The base-calling and quality-calling for Sanger sequences were inferred from the .ab1 raw chromatogram files using 'phred' [20,21] and the sequences were quality-trimmed using 'lucy' [22]. MIRA assembler (v 3.2.0) was used to build an assembly of the V.test genome with the following flags and options: "-job = denovo, genome, accurate, sanger, 454 -highlyrepetitive -AS:klrs = no 454_SETTINGS -AS:urdcm = 1.1:ardml = 100''. This assembly yielded a very large contig containing a complete prDNA unit, and a second contig containing an incomplete unit bearing the prDNA/prDNA junction. The complete prDNA unit was extracted from the first contig and identified as being the last prDNA unit before the LUR junction and noted prDNA-G following Bublot *et al*. [14]. By analysing the contig bearing the prDNA/prDNA junction in GAP4 [23], we determined a 518 bp fragment of the prDNA-inner unit (as named by Bublot *et al*. [14]) bordered on the left by lower read qualities and coverage, and on the right by the beginning of a new prDNA unit. This end was joined to the beginning (2,089 bp) of the prDNA-G unit in order to obtain a complete prDNA-inner unit (2,607 bp). We verified that this complete unit was compatible with previously published information [14].

### BoHV-4 genome annotation

All Open Reading Frames (ORFs) from all 6 frames were retrieved from the complete genomic sequence and matched against the Conserved Domain Database [24] using the position-specific scoring matrices (PSSM) based Reverse PSI-BLAST [25]. For all ORFs sharing the same STOP and containing a PSSM match, the smallest ORF containing the largest PSSM match was retained. 59 ORFs were thus considered evolutionarily conserved and were annotated with the corresponding matching conserved domains. Out of the 79 CDS from the previously published 66-p-347 strain, all 59 ORFs matched previously annotated 66-p-347 ORFs. The 20 remaining CDS were added by similarity to this strain and were annotated as such. Repeat segments and special features were annotated according to 66-p-347 if they were present in V.test. The complete genome sequence containing the LUR, prDNA-G and prDNA-inner were annotated and submitted to GenBank with respective accession numbers: JN133502, JN133503 and JN133504.

### Comparative genomics analysis of 66-p-347 and V.test

The LUR and prDNA sequences of the 66-p-347 strain were joined into a complete genome (accession numbers NC_002665 and AF092919) and aligned against the joined LUR and prDNA-inner V.test sequences with ClustalW 2.0.10 [26]. Percent divergence, percent insertions and deletions, and percent G+C content were computed (i) along the alignment on a 100 bp sliding window of step 3 bp and (ii) on all individually aligned proteins. Analyses and figures were conducted using R [27] and the seqinr [28] package in combination with *ad hoc *programs written in Python and using the Biopython libraries [29,30].

### RT-PCR analysis

These experiments were performed as described elsewhere [31]. Briefly, subconfluent monolayers of MDBK cells were infected with BoHV4 V.test strain at a m.o.i. of 1 PFU/cell. 18 hours after infection, cytoplasmic RNA was extracted, purified and treated for RT-PCR. The cDNA products were amplified by PCR using specific primers listed in Table 1.

## Results and discussion

### BAC sequencing and genome assembly

Pyrosequencing of herpesviral genomes is often limited by the high concentration of contaminating cellular DNA [32]. We therefore prepared the BoHV-4 V.test strain DNA from BAC maintained genomes and sequenced it using a high-throughput pyrosequencing approach [16]. This yielded 48,967 reads among which 47,800 were BoHV-4 specific (> 97% of the reads). After assembly, the mean genome coverage was of the order of 96×. In comparison to the whole genome sequencing of another herpesvirus based on DNA isolated from virus particles, which exhibited a 13× average base pair coverage [32], our strategy showed a more than 7-fold increase. This is probably mainly due to the high proportion of viral to cellular reads present in our dataset. Indeed, only 1,167 *Escherichia coli *contaminant reads had to be discarded from the data, indicating less than 2.38% of contaminated reads, compared to the previously reported 62.72% contaminating cellular reads in [32]. Our sequencing strategy based on a BAC cloning approach, thus revealed itself very powerful in terms of contamination and subsequent coverage.

### V.test genome analysis and comparison to other BoHV-4 strains

**Figure 1:**
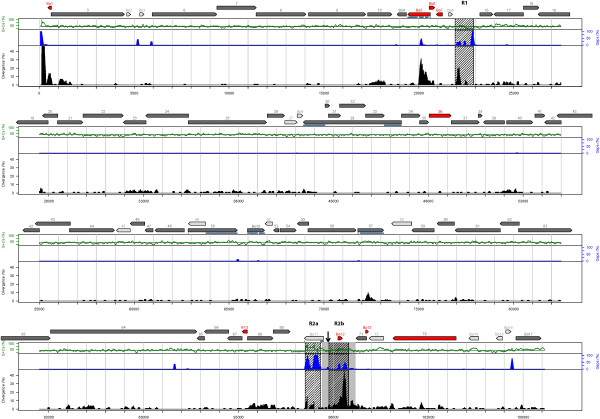
**Map of the BoHV-4 V.test strain genome and divergence with the 66-p-347 strain sequence**. The LUR of both strains have been aligned. Genome features are represented in the upper part as grey and red oriented arrows. Red arrows represent genes with an in-frame STOP codon, an early Methionine or a high divergence level in the V.test strain compared to 66-p-347. Dark (resp. light) grey arrows represent genes with (resp. without) an evolutionarily conserved domain (see Methods). The exons of spliced genes are indicated under the given gene as thin light-blue lines. Percent divergence is shown as a black-filled curve, percent insertions and deletions are shown as a blue-filled curve. Percent G+C content is shown as a thin green curve, with the mean G+C content drawn as a thin horizontal green line. These percentages are measured in a 100 bp window sliding 3 bp. Repeat regions (R1, R2a, R2b) are depicted as hatched areas. The *oriLyt *region is mapped as a light-grey area within R2b and the conserved quasi-palindromic motif in the *oriLyt *region is indicated by a small vertical arrow.

The BoHV-4 genome has a B-type structure consisting of a long unique region (LUR) flanked by several polyrepetitive DNA units (prDNA). We assembled the complete LUR of the V.test strain BoHV-4 genome into a 108,241 bp sequence. The average G+C content is of 41.21%. This value as well as the G+C% variation observed on Figure 1 is in agreement with previously reported results on the 66-p-347 strain, namely on the high G+C content of R2a region [11]. The observed-to-expected CpG ratio is of 0.225 on the LUR and is compatible with the value measured on *Bos taurus *(0.234) [33] suggesting (i) a high degree of methylation of CpG nucleotides and (ii) similar methylation mechanisms acting on the viral and cellular genome.

As expected, the nucleotide identity between our assembled genome and previously published V.test strain sequence data was of 99.55% in average, falling into the ranges of comparison between 454 and Sanger sequencing [34].

Compared to the 66-p-347 strain, the V.test strain had previously shown divergence up to 12% on the region surrounding BORFB2 (ORF 16, v-Bcl-2) [11]. However, the lack of a complete genomic sequence for the V.test strain prevented from drawing a general conclusion concerning this divergence level. Compared to 66-p-347 strain, the overall V.test nucleotide identity is high (99.1%), but shows a large variability at the genome level (Figure 1). As expected, the repetitive regions contained in the LUR (R1, R2a and R2b) exhibit a high nucleotide divergence, up to more than 40%, as well as large gaps (Figure 1). This indicates that the very high divergence levels seem confined to specific repetitive genomic regions. However, some rather high divergence levels were also identified in other regions (Figure 1) and namely in ORF-containing regions such as ORF 10, Bo5, ORF 57, and ORF 68 region. We also note a large deletion and a high divergence at the beginning of the LUR compared to the 66-p-347 strain. Overall, these differences in protein-coding region as well as in repetitive regions that bear predicted microRNA coding sequences [35] will require specific experiments to identify possible links with observed phenotypic differences between strains.

### Conserved protein-coding genes

In order to develop an *ab initio *approach of gene annotation, we extracted all possible ORFs in all 6 frames from the complete genomic sequence of the BoHV-4 V.test strain. On each of these ORFs, we ran a Reverse PSI-BLAST [25] against all protein domains from the Conserved Domain Database [24]. ORFs containing an evolutionarily conserved domain were defined as the smallest ORF containing the longest CDD match (see Methods). This approach revealed 59 ORFs containing a conserved CDD domain (Table 2). All 59 detected ORFs corresponded to ORFs previously annotated in the 66-p-347 strain (on a total of 79 ORFs listed in Table 3), indicating that 75% of BoHV-4 ORFs contain conserved domains. Most of these ORFs (37/59) contain domains that are either conserved at different levels in the Herpesvirales (either gammaherpesvirinae, herpesviridae or herpesvirales), or at a much larger scale that include Eukaryota, Bacteria and Archaea (22/59) (Table 2). This second set of genes might bear good candidates for genes having been the stage of lateral gene transfer events as observed for several herpesvirus genes [36] such as the BoHV-4 Bo17 gene that encodes a homologue of the cellular core 2 beta -1,6-N-acetylglucosaminyl-transferase M [37]. These results will deserve further studies to identify the evolutionary history responsible for these observations.

**Table 2:** Potential BoHV-4 V.test ORFs presenting conserved functional domains

BoHV-4 ORF	Domain accession number	Functional annotation	**Species distribution**^**a**^
ORF 3	COG0046, TIGR01735, TIGR01736, TIGR01739, PF02769	Phosphoribosylformylglycinamidine (FGAM) synthase; AIR synthase related protein	Eukaryota; Bacteria; Archaea; Viruses
ORF 6	PF00747	ssDNA binding protein	Herpesviridae
**ORF 7**	**PF01366**	**Protein transporter activity**	**Herpesviridae**
**ORF 8**	**PF00606**	**Surface glycoprotein**	**Herpesviridae**
ORF 9	COG0417, TIGR00592, PF00136, PF03104, SM00486	DNA polymerase type-B family	Eukaryota; Bacteria; Archaea; Viruses
ORF 10	PF04797	dUTPase	Herpesviridae
Bo4	COG5183, SM00744	Protein involved in mRNA turnover and stability; zinc-finger RING-variant domain	Eukaryota; Bacteria
ORF 16	SM00337	BCL (B-Cell lymphoma); contains BH1, BH2 regions	Eukaryota; Bacteria; Viruses
**ORF 17**	**PF00716**	**Serine-type endopeptidase activity**	**Herpesviridae**
ORF 18	PF03049	UL79 family	Herpesviridae
**ORF 19**	**PF01499**	**Virus penetration and capsid assembly**	**Herpesviridae**
**ORF 20**	**PF01646**	**UL24 family**	**Herpesviridae**
ORF 21	PF00693, PF01712, PF08465	ATP binding; thymidine kinase activity; phosphotransferase activity	Eukaryota; Bacteria; Viruses
**ORF 22**	**PF02489**	**Virion associated envelope glycoprotein**	**Herpesviridae**
ORF 23	PF04682	BTRF1 protein conserved region	Herpesviridae
ORF 24	PF03043	UL87 family	Herpesviridae
ORF 25	PF03122	Structural molecule activity	Herpesvirales
**ORF 26**	**PF01802**	**Structural molecule activity**	**Herpesvirales**
ORF 29	PF02499, PF02500	Probable role in DNA packaging	Herpesvirales
ORF 30	PF05338	Unknown function (DUF717)	Gammaherpesvirinae
ORF 31	TIGR01234, PF03048	UL92 family; L-ribulokinase	Herpesvirales; Embryophyta
ORF 32	PF04559	DNA cleavage and packaging	Herpesviridae
ORF 33	PF03044	Possible role in capsid maturation	Herpesviridae
ORF 34	PF03038	UL95 family	Herpesviridae
ORF 35	PF05852	Unknown function (DUF848)	Gammaherpesvirinae
**ORF 36**	**COG0515, PF00069, SM00220**	**ATP binding; protein kinase activity; Serine/threonine protein kinase**	**Eukaryota; Bacteria; Archaea**
**ORF 37**	**PF01771, PF09588**	**Exonuclease activity; DNA binding**	**Eukaryota; Bacteria; Viruses**
ORF 38	PF10813	Unknown function (DUF2733)	Herpesviridae
**ORF 39**	**PF01528**	**Integral membrane protein**	**Herpesviridae**
ORF 40	PF03324	Helicase-primase complex associated protein	Herpesviridae
ORF 41	PF05774	Helicase-primase complex components	Gammaherpesvirinae
**ORF 42**	**PF01677**	**UL7-like protein**	**Herpesviridae**
**ORF 43**	**PF01763**	**Possible role in cleavage and packaging**	**Herpesviridae**
**ORF 44**	**COG0507, PF02689**	**Helicase; ATP-dependent exoDNAse (exonuclease V)**	**Eukaryota; Bacteria; Viruses**
**ORF 46**	**COG0692, TIGR00628, TIGR03443, PF03167**	**Uracil DNA glycosylase**	**Eukaryota; Bacteria; Archaea; Viruses**
ORF 47	PF11108	Glycoprotein L	Herpesviridae
ORF 48	PF05734	Unknown function (DUF832)	Herpesviridae
ORF 50	PF03326, PF04793	Early-intermediate transcription factors	Gammaherpesvirinae
**Bo10**	**PF05459, PF05812**	**Transcriptional regulator proteins**	**Herpesviridae**
ORF 53	PF03554	Highly polymorphic glycoprotein	Herpesviridae
**ORF 54**	**COG0756, TIGR00576, PF00692**	**dUTPase**	**Eukaryota; Bacteria; Archaea; Viruses**
ORF 55	PF04533	U44 protein	Herpesviridae
**ORF 56**	**PF03121**	**UL52/UL70 DNA primase**	**Eukaryota; dsDNA Viruses**
ORF 57	PF04633	BMRF2 protein	Gammaherpesvirinae
ORF 59	PF04929	DNA replication accessory factor	Gammaherpesvirinae
ORF 60	COG0208, PF00268	Ribonucleotide reductase	Eukaryota; Bacteria; Archaea; Viruses
**ORF 61**	**COG0209, TIGR02504, TIGR02506, TIGR02510, PF00317, PF02867**	**Ribonucleotide reductase**	**Eukaryota; Bacteria; Archaea; Viruses**
ORF 62	PF03327	Capsid shell protein VP19C	Herpesviridae
ORF 63	PF04523	Tegument protein U30	Herpesviridae
ORF 64	PF04843	Tegument protein, N-terminal conserved region	Herpesviridae
ORF 65	PF06112	Capsid protein	Gammaherpesvirinae
ORF 66	PF03117	UL49 family	Herpesviridae
ORF 67	PF04541	Virion protein U34	Herpesviridae
ORF 67.5	PF03581	UL33-like protein	Herpesviridae
**ORF 68**	**PF01673**	**Putative major envelope glycoprotein**	**Herpesviridae**
**ORF 69**	**PF02718**	**UL31-like protein**	**Herpesviridae**
ORF 71	PF01335, SM00031	Death effector domain	Eukaryota; Viruses
ORF 75	COG0046, COG0047, TIGR01735, TIGR01736, TIGR01739, TIGR01857, PF02769	Phosphoribosylformylglycinamidine (FGAM) synthase; AIR synthase related protein	Eukaryota; Bacteria; Archaea; Viruses
Bo17	PF02485	Core-2/I-Branching enzyme	Eukaryota; Bacteria; Viruses

Conserved functional domains were determined for each BoHV-4 V.test ORF using METHOD and the domains accession numbers, functional annotation and the species distribution were listed. When several domains were conserved, the annotations were either merged when possible or juxtaposed. Domains present in herpesviridae conserved gene families are highlighted in bold (*from Fu, 2008*). (PFxxx: Pfam Accession Number; SMxxx: SMART Accession Number; COGxxx: COG Accession Number; TIGRxxx: TIGRFam Accession Number).

^a ^Major taxonomic groups.

**Table 3:** Potential BoHV-4 V.test ORFs and homologues to HHV-8 and HHV-1

BoHV-4 ORF	Strand	**Start**^**a**^	**Stop**^**a**^	**HHV-8 homologue**^**b**^	**HHV-1 homologue**^**c**^	Annotation and comments
Bo1	-	272	460	*--*	*--*	Early in-frame STOP codon
***ORF 3***	***+***	***441***	***4307***	***--***	***--***	***BORFA1; v-FGAM-synthase***
Bo2	+	4435	4638	*--*	*--*	
Bo3	+	5072	5299	*--*	*--*	
***ORF 6***	***+***	***5703***	***9107***	***ORF 6***	***UL 29***	***single-stranded DNA-binding protein MDBP***
**ORF 7**	**+**	**9112**	**11190**	**ORF 7**	**UL 28**	**transport protein**
**ORF 8**	**+**	**11180**	**13804**	**ORF 8**	**UL 27**	**glycoprotein B**
***ORF 9***	***+***	***13944***	***16961***	***ORF 9***	***UL 30***	***DNA polymerase***
***ORF 10***	***+***	***17057***	***18337***	***ORF 10***	***--***	***BORFB1***
***Bo4***	***-***	***18604***	***19101***	***--***	***--***	***short ORF of immediate early transcript 1***
Bo5	-	19170	20355	K5	--	long ORF of immediate early transcript 1
Bo6	+	20297	20590	--	--	Early in-frame STOP codon
Bo7	-	20670	20994	--	--	Disrupted frame
Bo8	+	21318	21521	--	--	overlapping with late 1.7 kb RNA
***ORF 16***	***+***	***22967***	***23647***	***ORF 16***	***--***	***BORFB2; v-Bcl-2 protein***
**ORF 17**	**-**	**23710**	**25260**	**ORF 17**	**UL 26**	**capsid protein**
***ORF 18***	***+***	***25259***	***26077***	***ORF 18***	***--***	
**ORF 19**	**-**	**26029**	**27705**	**ORF 19**	**UL 25**	**tegument protein**
**ORF 20**	**-**	**27407**	**28219**	**ORF 20**	**UL 24**	
***ORF 21***	***+***	***28203***	***29540***	***ORF 21***	***UL 23***	***thymidine kinase***
**ORF 22**	**+**	**29551**	**31674**	**ORF 22**	**UL 22**	**glycoprotein H**
***ORF 23***	***-***	***31671***	***32873***	***ORF 23***	***--***	
***ORF 24***	***-***	***32851***	***35109***	***ORF 24***	***--***	
***ORF 25***	***+***	***35099***	***39220***	***ORF 25***	***UL 19***	***major capsid protein***
**ORF 26**	**+**	**39256**	**40170**	**ORF 26**	**UL 18**	**capsid protein**
ORF 27	+	40184	40829	ORF 27	--	
Bo9	+	40831	41130	*--*	*--*	
***ORF 29***	***-***	***41154***	***46328***	***ORF 29***	***UL 15***	***cleavage/packaging protein***
***ORF 30***	***+***	***42306***	***42548***	***ORF 30***	***--***	
***ORF 31***	***+***	***42482***	***43123***	***ORF 31***	***--***	
***ORF 32***	***+***	***43069***	***44439***	***ORF 32***	***UL 17***	***viral DNA cleavage/packaging protein***
***ORF 33***	***+***	***44432***	***45430***	***ORF 33***	***UL 16***	
***ORF 34***	***+***	***46327***	***47313***	***ORF 34***	***--***	
***ORF 35***	***+***	***47285***	***47758***	***ORF 35***	***--***	
**ORF 36**	**+**	**47787**	**48950**	**ORF 36**	**UL 13**	**kinase**
**ORF 37**	**+**	**48958**	**50427**	**ORF 37**	**UL 12**	**alkaline exonuclease**
***ORF 38***	***+***	***50379***	***50585***	***ORF 38***	***--***	
**ORF 39**	**-**	**50652**	**51761**	**ORF 39**	**UL 10**	**glycoprotein M**
***ORF 40***	***+***	***51877***	***53247***	***ORF 40***	***UL 8***	***helicase-primase complex component***
***ORF 41***	***+***	***53360***	***53881***	***ORF 41***	***--***	***helicase-primase complex component***
**ORF 42**	**-**	**53873**	**54748**	**ORF 42**	**UL 7**	
**ORF 43**	**-**	**54525**	**56375**	**ORF 43**	**UL 6**	**capsid protein**
**ORF 44**	**+**	**56323**	**58701**	**ORF 44**	**UL 5**	**helicase**
ORF 45	-	58805	59530	ORF 45	--	
**ORF 46**	**-**	**59530**	**60291**	**ORF 46**	**UL 2**	**uracil-DNA-glycosidase**
***ORF 47***	***-***	***60309***	***60731***	***ORF 47***	***--***	***glycoprotein L***
***ORF 48***	***-***	***60838***	***62382***	***ORF 48***	***--***	
***ORF 50***	***+***	***62586***	***65179***	***ORF 50***	***--***	***immediate early transcript 2; R transactivator protein***
ORF 49	-	62600	63499	ORF 49	--	
**Bo10**	**+**	**65696**	**66595**	**--**	**UL 54**	**glycoprotein gp80**
ORF 52	-	66621	67007	ORF 52	--	
***ORF 53***	***-***	***67073***	***67345***	***ORF 53***	***--***	
**ORF 54**	**+**	**67414**	**68262**	**ORF 54**	**UL 50**	**dUTPase**
***ORF 55***	***-***	***68321***	***68923***	***ORF 55***	***--***	
**ORF 56**	**+**	**68887**	**71418**	**ORF 56**	**UL 52**	**DNA replication protein**
***ORF 57***	***+***	***71512***	***72870***	***ORF 57***	***--***	***possible post-transcriptional transactivator***
ORF 58	-	73294	74346	ORF 58	--	
***ORF 59***	***-***	***74360***	***75535***	***ORF 59***	***--***	***DNA replication protein***
***ORF 60***	***-***	***75688***	***76605***	***ORF 60***	***UL 40***	***ribonucleotide reductase small subunit***
**ORF 61**	**-**	**76639**	**79020**	**ORF 61**	**UL 39**	**ribonucleotide reductase large subunit**
***ORF 62***	***-***	***79002***	***80021***	***ORF 62***	***UL 38***	***assembly/DNA maturation protein***
***ORF 63***	***+***	***79978***	***82797***	***ORF 63***	***--***	***tegument protein***
***ORF 64***	***+***	***82812***	***90488***	***ORF 64***	***UL 36***	***tegument protein***
***ORF 65***	***-***	***90504***	***90896***	***ORF 65***	***--***	***capsid protein***
***ORF 66***	***-***	***90893***	***92167***	***ORF 66***	***--***	
***ORF 67***	***-***	***92107***	***92877***	***ORF 67***	***UL 34***	***tegument protein***
***ORF 67.5***	***-***	***92881***	***93144***	***ORF 67.5***	***UL 33***	***Disrupted (late) methionine***
**ORF 68**	**+**	**93155**	**94504**	**ORF 68**	**UL 32**	**probable glycoprotein**
**ORF 69**	**+**	**94512**	**95405**	**ORF 69**	**UL 31**	
Bo11	-	96158	96703	--	--	
Bo12	+	97442	97684	--	--	Early in-frame STOP codon
***ORF 71***	***-***	***98328***	***98876***	***K13/ORF 71***	***--***	***BORFE2; v-FLIP***
Bo13	+	98838	98983	--	--	Disrupted frame
ORF 73	-	99022	99783	ORF 73	--	BORFE3, LANA homologous
***ORF 75***	***-***	***100274***	***103606***	***ORF 75***	***--***	***Disrupted (late) methionine; tegument protein/v-FGAM-synthetase***
Bo14	-	104273	104785	*--*	*--*	
Bo15	-	105724	106038	*--*	*--*	
Bo16	+	106225	106494	*--*	*--*	
***Bo17***	***+***	***106681***	***108003***	***--***	***--***	***viral beta-1,6-N-acetylglucosaminyltransferase***

^a ^Positions of the respective ORFs of BoHV-4 on the LUR sequence. These are given from the first nucleotide of the start codon ATG to the last nucleotide of the stop codon.

^b ^Correspondance to HHV-8 genes is according to Zimmermann (2001).

^c ^Correspondance to HHV-1 genes was based on the presence of conserved domain with a BoHV-4 gene. Genes containing evolutionary conserved domains are highlighted in bold italic. The genes conserved in Herpesviridae are highlighted in bold (*from Fu, 2008*).

### Non-conserved protein-coding genes

**Figure 2:**
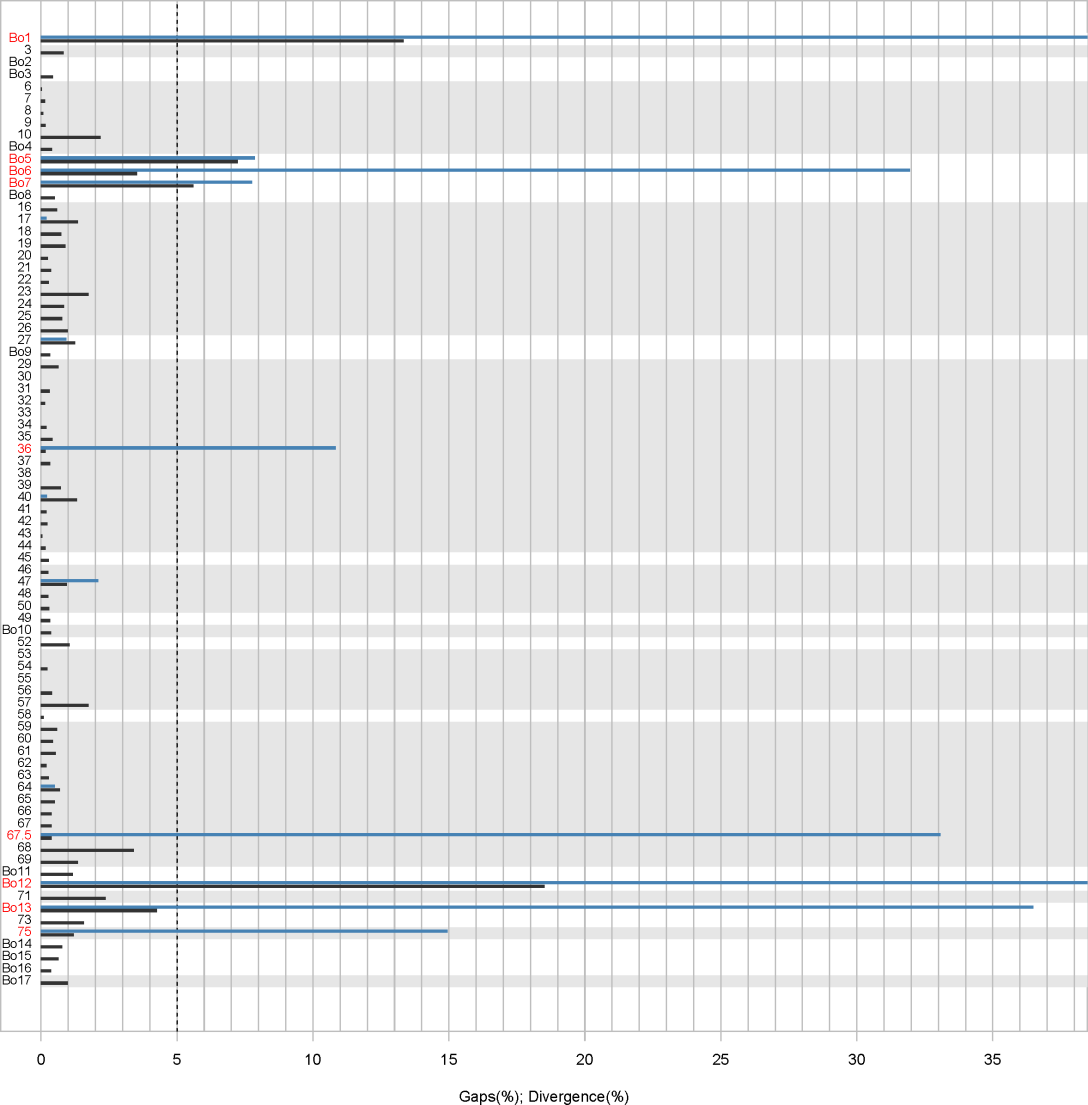
**Proteins in V.test: divergence with the previously published 66-p-347 strain**. Percentage divergence and percentages indels on the aligned amino-acid sequences are represented as, respectively, black and light-blue bars. The dotted black line represents a 5% threshold. Genes containing an evolutionarily conserved domain (see Methods) are represented on a light-grey background. Previously annotated genes presenting in the V.test strain an in-frame stop codon, a late Methionine or large divergence levels compared to the 66-p-347 strain are indicated in red.

The remaining 20 annotated ORFs were determined by similarity to the 66-p-347 strain, and correspond for most of them to ORFs unique to BoHV-4 as described previously (Table 3) [11]. Some of these ORFs, however, contain odd characteristics that needed to be investigated (Figure 2, Additional file 1 Figures S1-9). Indeed Bo1, Bo6, Bo7, Bo12 and Bo13 genes of the BoHV-4 V.test strain present in-frame STOP codons. Bo5 presents rather high divergency levels and large insertions/deletions (> 5% of its coding sequence as shown in Figure 2) compared to the genomic sequence of the 66-p-347 strain. Moreover, ORFs 36, 67.5 and 75, which bear an evolutionary conserved domain, present late methionines compared to the 66-p-347 annotation. Indeed, in ORF 36 (see Additional file 1 Figures S5), the smallest ORF containing an evolutionary conserved domain is slightly shorter than the one annotated in 66-p-347 and there is no evidence that the previously annotated methionine is the correct one. However, comparison with homologous genes in other rhadinoviruses suggests that the start codon proposed in the 66-p-347 annotated sequence is the most likely. In ORF 67.5 (see Additional file 1 Figures S6), there is a point substitution in the 66-p-347 annotated ATG leading to the identification of a subsequent ATG as the V.test methionine. Finally, ORF 75 presents a small phase-disrupting indel in its 5' end (see Additional file 1 Figures S9), leading to the absence of the 66-p-347 annotated methionine in the V.test strain. All these annotated genes requested therefore an investigation of their transcription in mRNA products.

**Figure 3:**
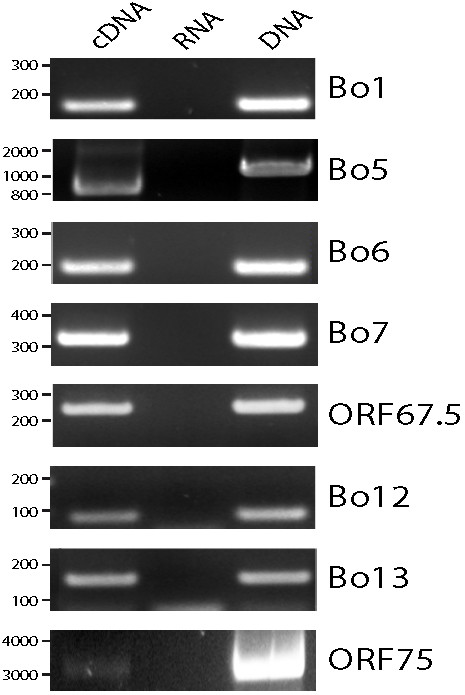
**RT-PCR amplification of the coding regions of the genes Bo1, Bo5, Bo6, Bo7, ORF67.5, Bo12, Bo13 and ORF75 of the BoHV-4 V.test strain**. Subconfluent monolayers of MDBK cells were infected with BoHV4 V.test strain at a m.o.i. of 1 PFU/cell. 18 hours after infection, cytoplasmic RNA was extracted, purified and treated for RT-PCR. The cDNA products were amplified by PCR using specific primers listed in Table 1.

As these sequence properties could be specific to the BAC clone of the BoHV-4 V.test strain, we investigated the transcription of these genes on MDBK cells infected with the BoHV-4 V.test WT strain as described in the methods. The primers used are described in Table 1 and highlighted in Additional file 1. For all couple of primers, cDNA from BoHV-4-infected MDBK cells gave rise to the expected PCR products (Figure 3). The absence of contaminant viral DNA in the mRNA preparations was confirmed by a lack of PCR product without reverse transcriptase. The size of the Bo5 RT-PCR product was also consistent with its known mRNA splicing (868 bp rather than 1140 bp). Moreover, the sequences of these RT-PCR products were in agreement with the BoHV-4 V.test sequence derived from our BAC cloned genome (data not shown). Therefore, we can conclude that all these coding sequences are transcribed during BoHV-4 infection of MDBK cells. However, further investigation is needed to determine the presence of proteins and ensure their accurate annotation.

### BoHV-4 V.test replication origin

**Figure 4:**
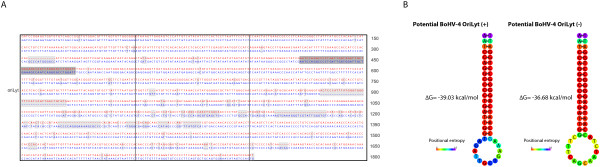
**Prediction of BoHV-4 V.test OriLyt**. A. Alignment of the V.test strain (above) and 66-p-347 strain (below) regions predicted to contain the OriLyt in the 66-p-347 strain. The differences observed in the alignment are highlighted in light grey. The predicted potential OriLyt is highlighted in dark grey. B. The predicted secondary structures of the top (+) and bottom (-) strands of the predicted BoHV-4 OriLyt sequence were analyzed using the Vienna RNA website program RNAfold with DNA parameters. The predicted free energy (ΔG) of each structure is given, as well as the positional entropy of each nucleotide.

A large region containing the potential lytic replication origin (*oriLyt*) of the BoHV-4 66-p-347 strain was determined by Zimmermann *et al *[11]. Based on this information, we mapped this region on the V.test genome (Figure 1). This region contains Bo12, the R2b region and partially overlaps with Bo11. Compared to the 66-p-347 strain sequence, the corresponding region in the V.test genome is highly divergent (Figure 4). Although this region shows high divergence rates, we expected the replication origin to be conserved between the two BoHV-4 strains. Previous work on other herpesviruses has identified in *oriLyt *the presence of palindromic motifs essential for viral replication [38-40]. When we compared the potential region containing *oriLyt *in the two strains, a single conserved palindromic region was observed (AATCCAGGCCCCTGATTGGTAGATTGCTGAAAGCCAATCAGGGGCCTGGATT, Figure 4). Interestingly, this region forms a perfect hairpin structure (Figure 4B) that resembles DNA structures formed at other herpesvirus origins [41,42] and may therefore represent a common secondary structure used by all herpesvirus family members during the initiation of DNA replication. In the future, this structure will be tested as a candidate for an essential *oriLyt *replication motif.

### BoHV-4 V.test polyrepetitive DNA

In the BAC clone, previous restriction profiles had determined a hypermolar prDNA band indicating that the BAC contained several prDNA units [12]. Therefore, the major pitfall in the assembly of the BoHV-4 V.test strain was the determination of the prDNA sequence. Indeed, (i) the higher per base coverage on this region due to repetition of prDNA units, (ii) the high GC content, along with (iii) the presence of several long repeats within the prDNA and (iv) the variability observed between prDNA units [14] made it extremely difficult to resolve and assemble with pyrosequencing data alone. Interestingly, it has been shown for several rhadinoviruses that the left junction between the prDNA and the LUR is the site of genome rearrangements and that sequences of the prDNA are found within the first base pairs of the LUR. These properties make this region very difficult to sequence [43-47].

Therefore, we adopted a hybrid strategy consisting in adding some ABI-Sanger reads (with the primers described in Table 1) to guide the 454 assembly on the prDNA region (see Methods).

Bublot, *et al*. [14] described the different prDNA unit variants present in BoHV-4 V.test, and namely the differences between prDNA units. Firstly, the prDNA units vary according to the number of repetitions of a ~200 bp Pst-I bordered fragment. Secondly, the last prDNA before the prDNA/LUR junction (prDNA-G) displays a different ending than the inner prDNA units [14]. Our method allowed us to disentangle the repeats and to assemble a contig containing a whole prDNA unit (2,440 bp) along with the left prDNA-LUR junction. This prDNA unit, corresponding to prDNA-G following Bublot *et al*. [14], was extracted from the contig and annotated. A second contig from this hybrid assembly yielded the prDNA/prDNA junction. The presence of the prDNA/prDNA junction in our assembly confirmed the presence of at least two prDNA units in our BAC clone and allowed us to build a complete prDNA-inner unit (see Methods). The assembled prDNA-G and inner prDNA units have sizes of 2,440 bp and 2,607 bp respectively. Both these units are in agreement with their previously published restriction maps [14].

**Figure 5:**
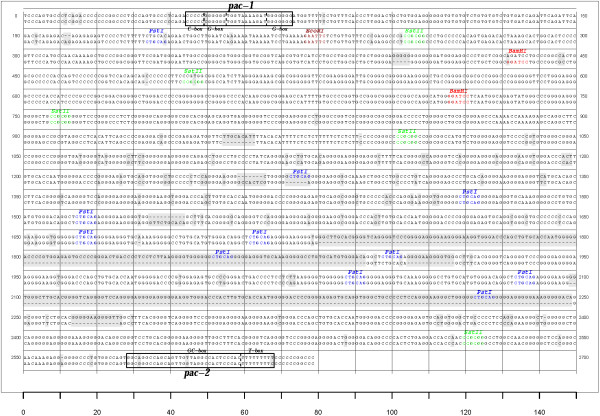
**The BoHV-4 inner prDNA units contain conserved cleavage/packaging signals**. Alignment of the prDNA-inner units from V.test strain (above) and 66-p-347 strain (below). The differences observed in the alignment are highlighted in grey. The cleavage/packaging signals *pac-1 *and *pac-2 *are represented in boxes, and their composing C-rich, G-rich, GC-rich and T-rich units are indicated. PstI, EcoRI, SstII, BamHI restriction sites are represented in coloured font.

Specifically, we showed that, comparatively to the 66-p-347 strain, the V.test prDNA-inner unit presents several indels including two large indels in the repetitive PstI region (Figure 5). This PstI-rich repetitive region seems to be the one presenting the most variation as it also presents comparatively large differences between prDNA units within the same strain. Indeed, Bublot *et al*. [14] roughly determined the size of the V.test major prDNA-inner unit to be around 2,650 bp due to the presence of 4 repetitions of the two small PstI bordered fragments.

**Figure 6:**
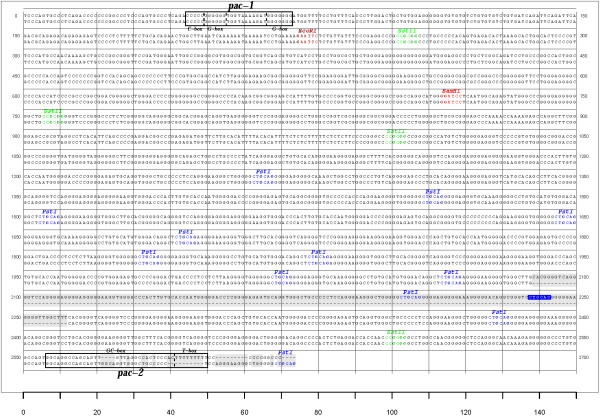
**The prDNA-G unit does not present a complete *pac-2 *cleavage/packaging signal**. Alignment of the prDNA units from V.test strain (prDNA-inner above and prDNA-G below). The differences observed in the alignment are highlighted in grey. The cleavage/packaging signals *pac-1 *and *pac-2 *are represented in boxes, and their composing C-rich, G-rich, GC-rich and T-rich units are indicated. PstI, EcoRI, SstII, BamHI restriction sites (here PstI) are represented in coloured font. One modified PstI restriction site in the prDNA-inner is also highlighted to indicate the divergence between the fragments composing both units.

In the prDNA-G unit, we established that these two small PstI-bordered fragments make up a fragment of 186 bp and that these are indeed repeated 4 times (Figure 6). In the prDNA-inner unit, we determined that the last PstI-bordered fragment is actually a variation of the 186 bp fragment where the inner Pst-I site is slightly modified (Figure 6). Therefore, the rough 200 bp size discrepancy between the prDNA-G (2,440 bp) and the prDNA-inner units (2,607 bp) is due to the presence of a slightly modified repetition of the previous segment. These results are compatible with the restriction profiles presented in Bublot *et al*. [14] as detailed by the positions of several restriction sites on Figure 6.

In addition to the variations in the PstI-bordered repetitions, one of the major differences between the prDNA-inner units and the prDNA-G lies in their 5' end. Indeed, the prDNA-inner contains a conserved *pac-2 *cleavage/packaging signal in its right terminal region, which is not the case of prDNA-G (Figure 6). Both units however, possess a conserved *pac-1 *cleavage/packaging signal in their left terminal region. Interestingly, the *pac-1 *and *pac-2 *cleavage and packaging signals show a good conservation between 66-p-347 and V.test's inner units, despite the presence of these signals in a repeated region bearing high divergence levels. Broll et al. [15] have determined, by transient cleavage/packaging assay, that a single prDNA unit is sufficient for cleavage and packaging. However, from the absence of a conserved *pac-2 *motif in the prDNA-G, we suggest that, even if a single inner prDNA unit is indeed sufficient for cleavage and packaging, the prDNA-G alone would not suffice. This would therefore indicate that two prDNA units at least are necessary in the context of naturally occurring BoHV-4 genomes for correct cleavage and packaging. The packaging of herpesvirus genomes is still not fully understood, however, detailed studies in herpes simplex virus type 1 (HSV-1), human and murine cytomegaloviruses (HCMV and MCMV) have highlighted the roles of the major conserved motifs and suggested the following general mechanism by which concatemers are cleaved and packaged [48-50]. Firstly, the T-box of the *pac-2 *signal is essential for the cleavage that initiates DNA packaging. Cleavage occurs at a fixed distance from the *pac-2 *T-box, and the resulting end that contains the *pac-2 *GC-box and other cis acting elements is inserted into the procapsid. Packaging is therefore directional and proceeds from *pac-2 *towards the *pac-1 *terminus [48]. A second cleavage event, directed by *pac-1*, then terminates DNA packaging. If we apply this model to BoHV-4, the divergence of the *pac-2 *signal in prDNA-G, namely the absence of a T-box, indicates that it is not a functional *pac-2 *initiation signal. As the genome packaging is directional from *pac-2 *to *pac-1 *(therefore, from the right to the left end of the genome), the lack of a *pac-2 *initiation signal in prDNA-G ensures that no packaging would lead to a remaining concatemer lacking a left end prDNA. This would therefore guarantee that genomes bearing at least one left and one right end prDNA unit are encapsulated into virions. This model and its implications will require further investigations in the future.

## Conclusions

BAC-cloning of the BoHV-4 V.test strain has greatly facilitated the use of this virus as a model for human pathogenic gammaherpesviruses. However, until now, the complete genome sequence of this strain was unavailable. In this study, we have determined the complete genome sequence of the BoHV-4 V.test strain. In comparison with the previously sequenced 66-p-347 strain, we identified important differences in 9 potential open reading frames. Moreover, sequence analyses allowed us to identify genome features that are potentially important for viral replication. All together, these results should have implications for the study of BoHV-4 and herpesviruses in general.

## Competing interests

The authors declare that they have no competing interests.

## Authors' contributions

LP analyzed the data in silico, participated in the RT-PCR assay and drafted the manuscript. BM prepared the viral DNA, participated in data analysis and performed the RT-PCR assay. AV and CL participated in data analysis. LG analyzed the data and drafted the manuscript. All authors read and approved the final manuscript.

## Supplementary Material

Additional file 1 **Alignments of the nucleotide and predicted amino acid sequences of Bo1 (Figure S1), Bo5 (Figure S2), Bo6 (Figure S3), Bo7 (Figure S4), ORF36 (Figure S5), ORF67.5 (Figure S6), Bo12 (Figure S7), Bo13 (Figure S8) and ORF75 (Figure S9) of BoHV-4 V.test and 66-p-347 strains**. Nucleotide sequences aligned at the amino-acid level are represented for BoHV-4 V.test (red) and 66-p-347 strains (blue). Mismatching residues are highlighted in a shaded grey box. The predicted amino-acid sequences are respectively drawn for V.test and for 66-p-347 above and below the nucleotide sequences. The STOP codons are highlighted by small colored boxes. The annotated Methionine are highlighted in bold font. In the Bo5 sequence, introns are represented by boxes. Positions of the specific primers used in Figure 3 are underlined. Click here for file
