# Supplementary material for: Sequencing of bovine herpesvirus 4 v.test strain reveals important genome features
Source: Virol J. 2011 Aug 16;8:406. doi: 10.1186/1743-422X-8-406 (PMC3178527; doi:10.1186/1743-422X-8-406)

Additional File 1, Figure S1

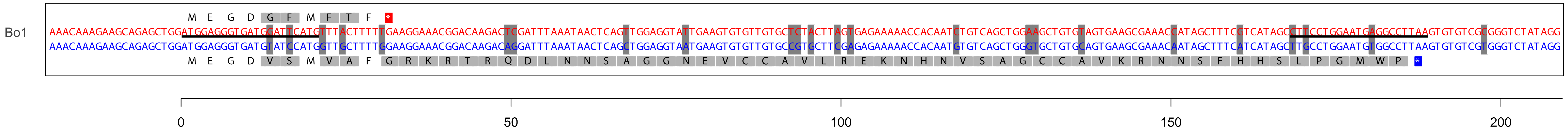

Additional File 1, Figure S2

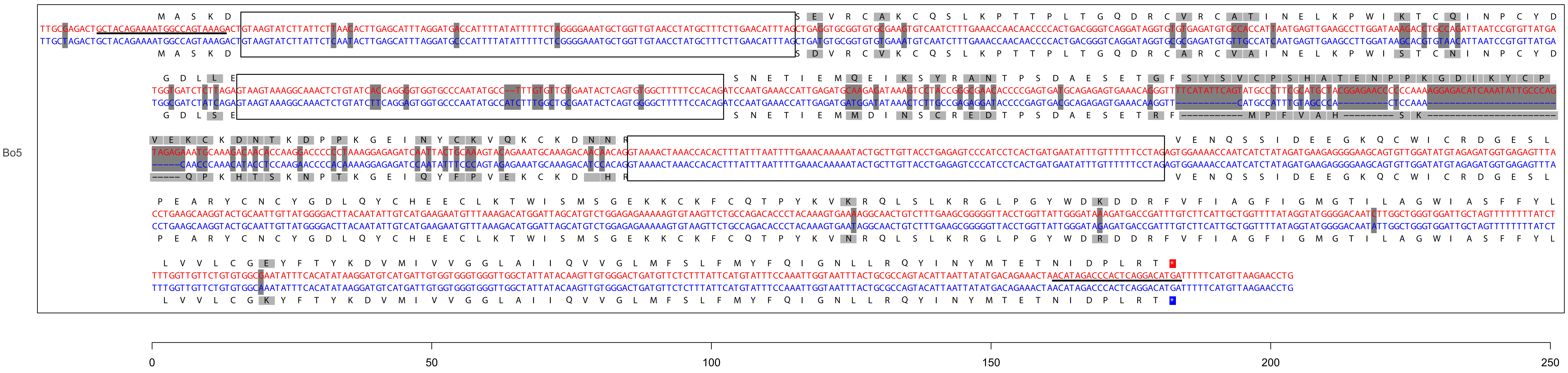

Additional File 1, Figure S3

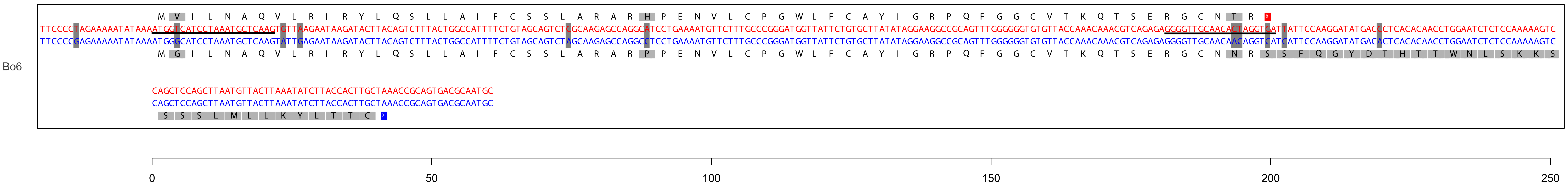

Additional File 1, Figure S4

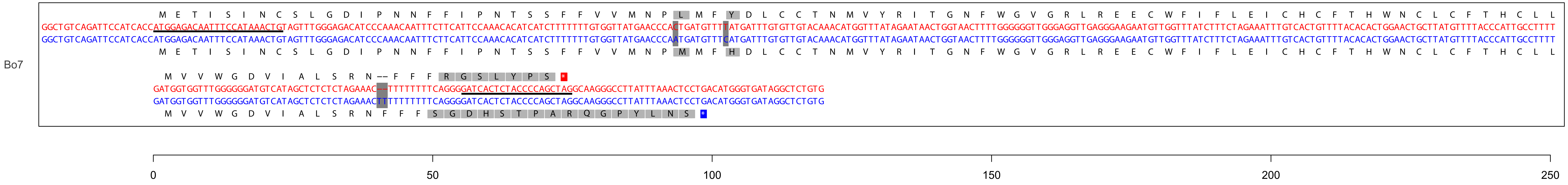

Additional File 1, Figure S5

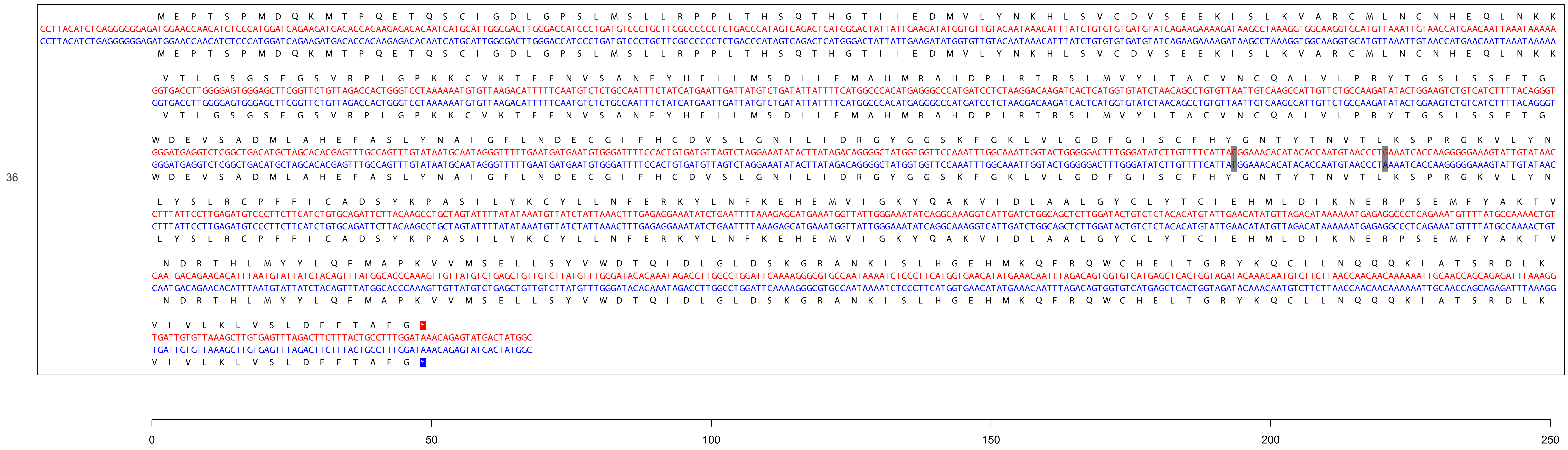

Additional File 1, Figure S6

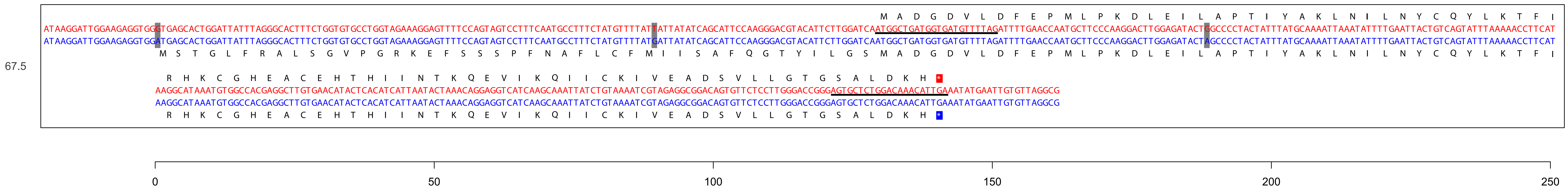

Additional File 1, Figure S7

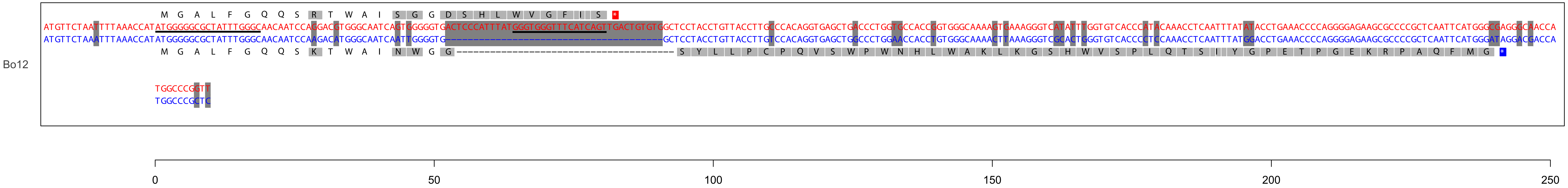

Additional File 1, Figure S8

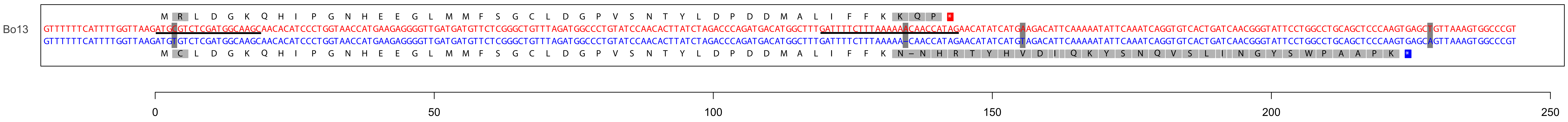

Additional File 1, Figure S9

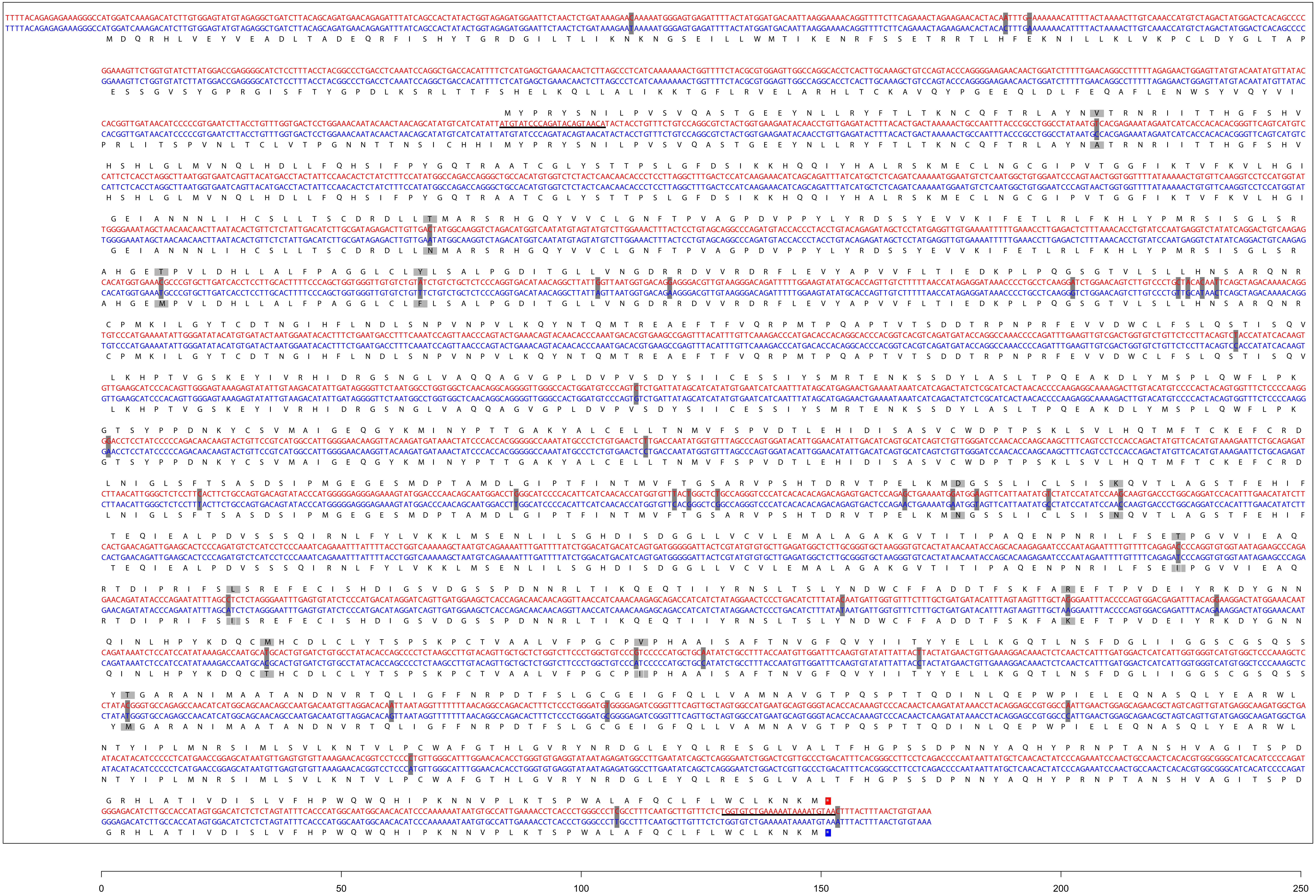

Supplement: Additional file 1 — Alignments of the nucleotide and predicted amino acid sequences of Bo1 (Figure S1), Bo5 (Figure S2), Bo6 (Figure S3), Bo7 (Figure S4), ORF36 (Figure S5), ORF67.5 (Figure S6), Bo12 (Figure S7), Bo13 (Figure S8) and ORF75 (Figure S9) of BoHV-4 V.test and 66-p-347 strains. Nucleotide sequences aligned at the amino-acid level are represented for BoHV-4 V.test (red) and 66-p-347 strains (blue). Mismatching residues are highlighted in a shaded grey box. The predicted amino-acid sequences are respectively drawn for V.test and for 66-p-347 above and below the nucleotide sequences. The STOP codons are highlighted by small colored boxes. The annotated Methionine are highlighted in bold font. In the Bo5 sequence, introns are represented by boxes. Positions of the specific primers used in Figure 3 are underlined. [file 1743-422X-8-406-S1.PDF]
